# Natural Selection Causes Adaptive Genetic Resistance in Wild Emmer Wheat against Powdery Mildew at “Evolution Canyon” Microsite, Mt. Carmel, Israel

**DOI:** 10.1371/journal.pone.0122344

**Published:** 2015-04-09

**Authors:** Huayan Yin, Yuval Ben-Abu, Hongwei Wang, Anfei Li, Eviatar Nevo, Lingrang Kong

**Affiliations:** 1 State Key Laboratory of Crop Biology, Shandong Key Laboratory of Crop Biology, College of Agronomy, Shandong Agricultural University, Tai’an, 271018, China; 2 Sapir Academic College, Projects and Physics Section, D.N. Hof Ashkelon, 79165, Israel; 3 Institute of Evolution, University of Haifa, 199 Aba Khoushy Ave., Mount Carmel, Haifa, 3498838, Israel; National Cancer Institute, UNITED STATES

## Abstract

**Background:**

“Evolution Canyon” (ECI) at Lower Nahal Oren, Mount Carmel, Israel, is an optimal natural microscale model for unraveling evolution in action highlighting the basic evolutionary processes of adaptation and speciation. A major model organism in ECI is wild emmer, *Triticum dicoccoides*, the progenitor of cultivated wheat, which displays dramatic interslope adaptive and speciational divergence on the tropical-xeric “African” slope (AS) and the temperate-mesic “European” slope (ES), separated on average by 250 m.

**Methods:**

We examined 278 single sequence repeats (SSRs) and the phenotype diversity of the resistance to powdery mildew between the opposite slopes. Furthermore, 18 phenotypes on the AS and 20 phenotypes on the ES, were inoculated by both *Bgt* E09 and a mixture of powdery mildew races.

**Results:**

In the experiment of genetic diversity, very little polymorphism was identified *intra*-slope in the accessions from both the AS or ES. By contrast, 148 pairs of SSR primers (53.23%) amplified polymorphic products between the phenotypes of AS and ES. There are some differences between the two wild emmer wheat genomes and the *inter*-slope SSR polymorphic products between genome A and B. Interestingly, all wild emmer types growing on the south-facing slope (SFS=AS) were susceptible to a composite of *Blumeria graminis*, while the ones growing on the north-facing slope (NFS=ES) were highly resistant to *Blumeria graminis* at both seedling and adult stages.

**Conclusion/Significance:**

Remarkable *inter*-slope evolutionary divergent processes occur in wild emmer wheat, *T*. *dicoccoides *at EC I, despite the shot average distance of 250 meters. The AS, a dry and hot slope, did not develop resistance to powdery mildew, whereas the ES, a cool and humid slope, did develop resistance since the disease stress was strong there. This is a remarkable demonstration in host-pathogen interaction on how resistance develops when stress causes an adaptive result at a *micro*-scale distance.

## Introduction

**Fig 1:**
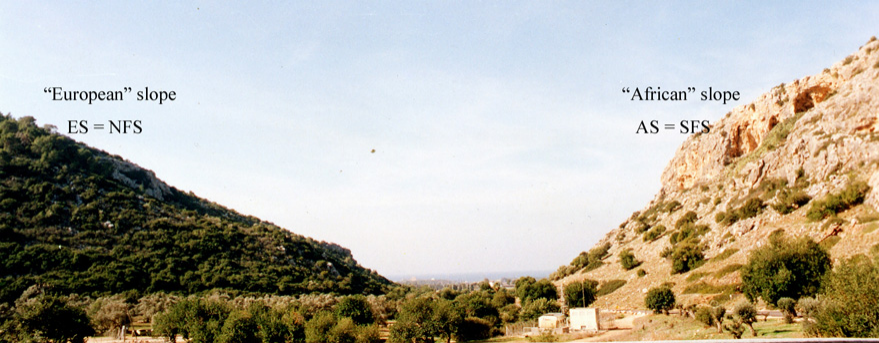
The “Evolution Canyon” I at Lower Nahal Oren, Mount Carmel, Isarel. Note the "African" savannah AS = SFS slope versus the forested ES = NFS slope. Higher terrestrial species richness occurs on the more stressful tropical AS. Aquatic species richness is higher on the milder, temperate cool and humid “European” slope (ES).

**Fig 2:**
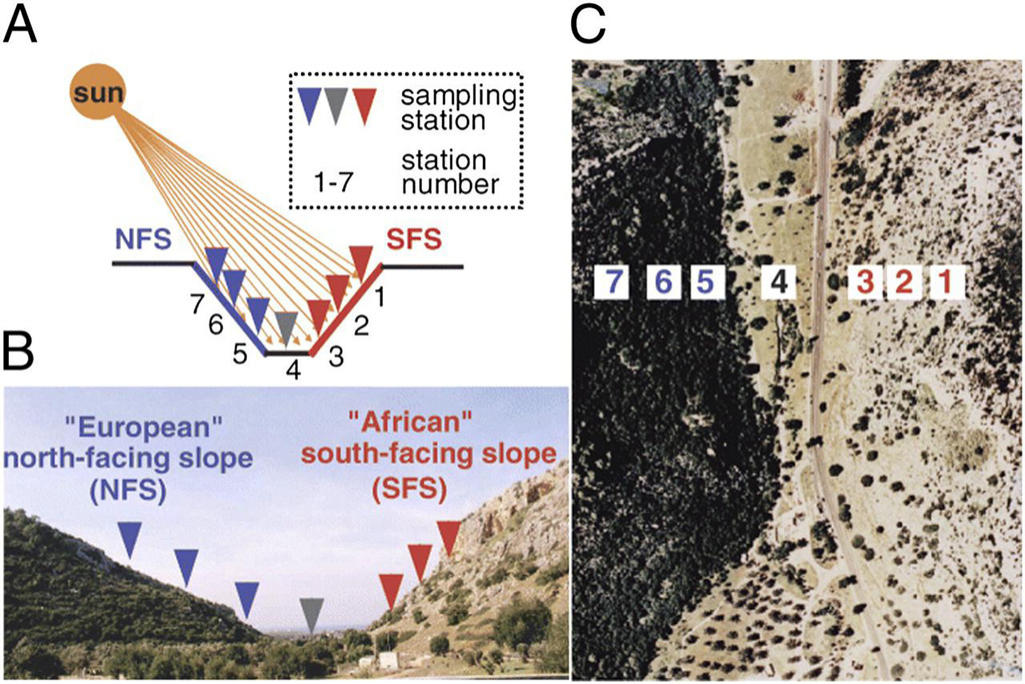
The EC model. A) Schematic diagram, B) Cross section view of "Evolution Canyon" I (EC I), Lower Nahal Oren, Mount Carmel, and C) Air view of EC I. Note the distinct divergent plant formations on the opposite slopes. The green, lush, European, temperate, cool-mesic, ES = NFS, sharply contrasts with the open park forest, warm-xeric, tropical, “African-Asian” savanna on the AS = SFS. In EC I, as in other three "Evolution Canyons" studied in Israel, seven sampling stations are designated: three on the AS = SFS (nos. 1–3), one at the valley bottom (no. 4) and three on the NFS (nos. 5–7). Source: Reference 2.

The "Evolution Canyon" (EC I) model is the subject of a long-term research program that started in 1990 at Lower Nahal Oren, Mount Carmel, Israel. EC I is an optimal natural *micro*-scale model for unraveling evolution in action highlighting the basic evolutionary processes of adaptation and speciation [1] (Fig 1 and Fig 2). It was expanded in three additional evolution canyons: EC II at Lower Nahal Keziv, westerm Upper Galilee; EC III at Nahal Shaharut, southern Negev Desert, and EC IV Mezar, southern Golan Heights) [2]. EC I, at Mount Carmel, studied here, (32°43′N; 34°58′E), consists of Upper Cenomanian limestones. Two thpusands and five hundred species have been identified in EC I, from bacteria to mammals in an area of 7,000 m. Solar radiation (up to 800% more) on the "African" (AS) south-facing slope (SFS) causes its tropical microclimate contrasting the "European" north-facing slope[2] (Fig 2). Higher terrestrial species richness was found on the more stressful tropical “African” slope (AS). Aquatic species richness was higher on the milder ES. By analyzing the genetic diversity between the AS and ES at Mount Carmel, Nevo et al [3] draw the following conclusions. First, microclimatic selection is the major evolutionary *inter*-slope, fast-acting, diverging force on genotypes and phenotypes, over-riding migration and genetic drift. Secondly, ecological stress can generate global-scale, adaptive evolutionary genome and phenome strategies at *micro*- and *macro*-scales, reinforcing homeostasis and fitness, and suggesting continuity between *micro*-evolution and *macro*-evolution.

Local microcosmic natural laboratories are dubbed “Evolution Canyon” (EC) models, where genomic, proteomic, and phenomic studies were carried out and focused on speciation and adaptation at a *micro*-scale. Speciation and adaptation are two important processes in studying evolution [2]. Viruses, bacteria, fungi, plants, and animals have been studied at the *micro*-scale involving biodiversity divergence, adaptation, and incipient adaptive ecological sympatric speciation to reveal evolution in action. Extensive studies have been conducted in "Evolution Canyon" in wild barley and other model organisms listed in Nevo "Evolution Canyon" publications at http://evolution.haifa.ac.il and in references [1–17]. These studies displayed dramatic interslope adaptive genomic divergence, slope-specific fitness components, and incipient adaptive ecological sympatric speciation on the opposite slopes.

The grass family Gramineae evolved 50–70 million years ago (Mya) [18, 19] and the sub-family Pooideae including wheat, barley, and oats had diverged around 20 Mya [20]. Wild diploid wheat (*T*. *urartu*, *2n* = 2X = 14, AA) hybridized with goat grass (*Aegilops speltoides*, *2n* = 2X = 14, BB) 300,000–500,000 BP to produce wild emmer wheat (*T*. *dicoccoides*, *2n* = 2X = 28, AABB) [21]. Wild emmer occurs in Israel, Syria, Lebanon, southeast Turkey, Jordan, northern Iraq, and western Iran. Natural populations of wild emmer have wide genotypic variations in agronomic, amino acid composition, protein quality and quantity, micronutrient contents, abiotic stress tolerances, herbicide resistances, and biotic stress tolerances [22–24].

Powdery mildew, caused by *Blumeria graminis* f. sp. *tritici (Bgt)*, is one of the devastating wheat diseases in areas with temperate climates. To date, several powdery mildew resistant genes (*Pm16*, *Pm26*, *Pm30*, *Pm36*, *MlZec 1*, *PmG3M*, *PmG16*, *Pm41*, *and Pm42*) are mapped using molecular markers in wild emmer [25–34].

Here we exhibited the patterns of 278 nuclear microsatellites and phenotypes on resistance to powdery mildew of wild emmer in “Evolution Canyon” (ECI), Lower Nahal Oren, Mt. Carmel, Israel. SSR sequences can serve functional roles as regulatory elements [35–40]. We revealed adaptive *inter*-slope divergence of the phenotype on resistance to powdery mildew between the tropical-xeric “African” slope and the temperate-mesic “European” slope.

## Materials and Methods

### Plant Materials

A total of 38 accessions of wild emmer were obtained from Eviatar Nevo from two stations (Stations 2 and 7) at the “Evolution Canyon” 1 (EC 1) in Mount Carmel. We can confirm that the field studies did not involve endangered or protected species. Israel Nature Reserve Authority responsible for Evolution Canyon. Israel Nature Reserve Authority issued permission for our study. Station 2 is on the south-facing slope, SFS, or “African” slope, AS, and station 7 is on the north-facing slope, NFS, or “European” slope, ES (Fig 2). The AS is richer in species of drought-tolerant taxa in the northern canyons, and the ES is richer in “humid” taxa, reflecting locally global patterns [1].

### Evaluation for Powdery Mildew Resistance

*Bgt* E09, a prevailing powdery mildew pathotype in China, virulent to *Pm1*, *Pm 3a*, *Pm3b*, *Pm3c*, *Pm3d*, *Pm3e*, *Pm3f*, *Pm5*, *Pm6*, *Pm7*, *Pm8*, *Pm17*, and *Pm19* [34], was used to inoculate 38 accessions (16 plants in each accession) at the seedling stage under controlled biological incubator conditions, 70% humidity and 18–20°C, in the winter of 2013. Huixianhong, a kind of highly susceptible common wheat, was used for the inoculation of seedlings. Disease reactions were recorded 9–13 days after inoculation, and infection types were scored on a 0–4 scale, with 0 representing no visible symptoms, 0; for necrotic flecks and 1, 2, 3, 4 for highly resistant, resistant, susceptible and highly susceptible reactions, respectively [27]. Phenotypes were pooled into two groups, resistant (R, IT = 0–2) and susceptible (S, IT = 3, 4). Furthermore, a mixture of powdery mildew races, collected in the experimental fields, was also used to inoculate 38 accessions under controlled greenhouse conditions at the seedling and adult stages. A randomized complete block design with three replications consisting of 1 m rows, with 20 plants at 5 cm spacing, 30 cm apart in greenhouse was used in this study.

### Genomic DNA Extraction and SSR Analysis

Total DNA was extracted from the healthy leaves of 38 accessions following the method of cetytrimethylammonium bromide (CTAB) [41] and there were also minor changes.

SSR markers mapped on the A and B genomes of hexaploid wheat ([42, 43]; http://www.graingenes.gov) were chosen for analysis of polymorphism between the wild emmer wheat genotypes from the AS and ES. We selected 278 SSR markers for testing (about 20 markers for each chromosome on average). 15μl volumes were taken in all PCR reactions containing 10mM Tris-Hcl, pH 8.3, 50mM Kcl, 1.5mM Mgcl_2_, 0.2mM dNTPs, 25 ng of each primer, 50-100ng genomic DNA and 0.75U Taq DNA polymerase. The amplification was performed at 94°C for 3 min, followed by 35 cycles at 94°C for 30s, at 55–62°C (depending on specific primers) for 30s, and at 72°C for 30s, with a final extension at 72°C for 10 min. SSR markers were performed on 8% non-denaturing polyacrylamide gels (39 acrylamide: 1 bisacrylamide). Gels were silver strained and photographed.

## Results and Discussion

**Fig 3:**
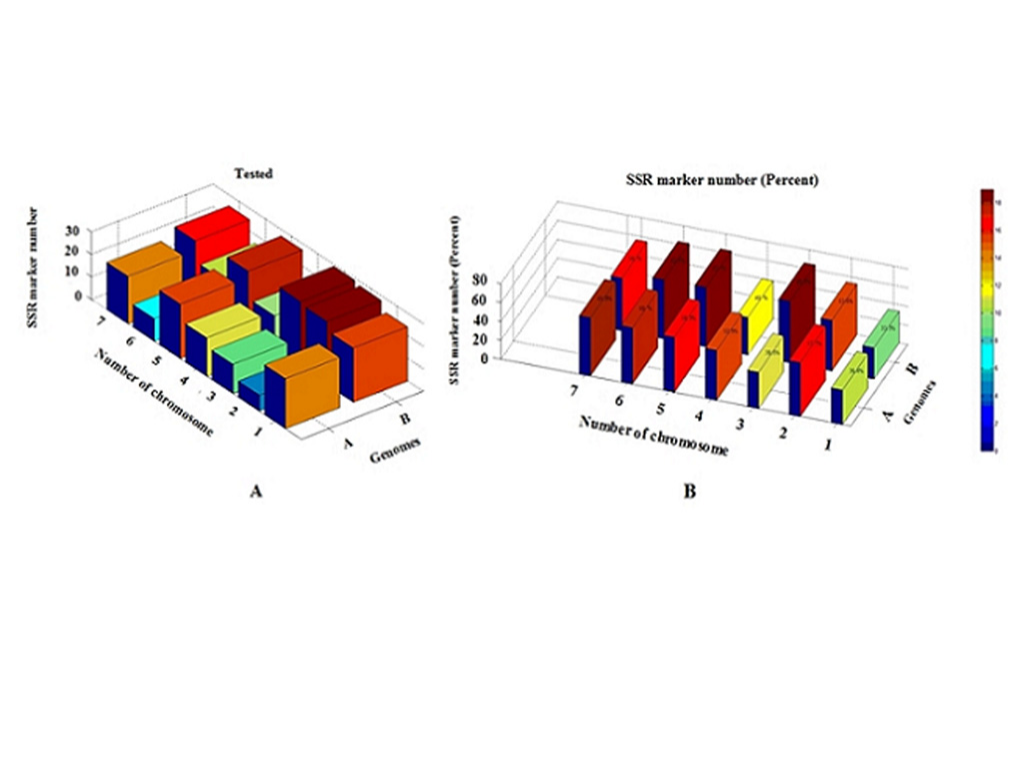
Interslope SSR polymorphism divergence in the A and B genomes of wild emmer wheat *Triticum dicoccoides*. A) Tested SSR markers between AS and ES on genomes A and B, B) SSR frequency polymorphism between AS and ES on the two genomes. Red color indicates high frequency and blue color low frequency.

A set of 278 wheat SSR markers located separately on 14 chromosomes were used to analyze the genetic diversity between the wild emmer wheat genotypes from the AS and ES (Table 1). Out of these, 114 SSR markers belong to genome A (1A-7A), and the other 164 SSR markers were distributed on genome B (1B-7B) (Table 1, Fig 3A). Interestingly, very little polymorphism was identified on the AS and ES *intra*-slope. Consequently, one phenotype from each slope, i.e. AS4 (from station 2; For AS4 genotype, total 127 pairs of SSR primers were screened, no polymorphic products were amplified on the AS *intra*-slope.) and a second ES7 (from station 7; Total 127 pairs of SSR primers were screened, for ES7 genotype, 2 of them amplified polymorphic products on the ES *intra*-slope.), were selected to represent AS genotypes and ES genotypes, respectively. Overall, 148 pairs of SSR primers (53.23%) amplified polymorphic products between the AS and ES (Table 1). We also used MATLAB 8.0 and Statistics Toolbox 8.1 software to illustrate the frequency distribution of genome A and B. The frequency polymorphism of SSR markers between AS and ES were similar on the genomes A and B, which are around 50% (Table 1, Fig 3B). This illustration amplify the differences between the two wild emmer wheat genomes and the *inter*-slope SSR polymorphic products between genome A and B. SSR sequences can serve functional roles as regulatory elements [35–40].

**Table 1:** Polymorphism investigated by SSR markers between AS and ES on 14 chromosomes.

	SSR markers					SSR markers			
Chromosome	Tested	Polymorphism between AS and ES			Chromosome	Tested	Polymorphism between AS and ES		
	N	N		%		N	N		%
**1A**	22	8	36.4		**1B**	24	8	33.3	
**2A**	7	4	57.1		**2B**	28	15	53.6	
**3A**	13	5	38.5		**3B**	29	19	65.5	
**4A**	17	9	52.9		**4B**	15	6	40	
**5A**	24	14	58.3		**5B**	27	17	63	
**6A**	10	6	60		**6B**	16	10	62.5	
**7A**	21	13	61.9		**7B**	25	14	56	
**Total**	114	59	51.2		**Total**	164	89	54.3	
**Mean**	16	8	50		**Mean**	23	13	56.5	

Remarkably, biodiversity evolution including functional ecological genomics diverges drastically on the abutting slopes across life from bacteria to mammals in biodiversity, adaptations and speciation patterns [1–17]. Microsites divergent sharply ecologically abiotically (by climate, rocks, soils, or chemicals), or biotically (parasites and pathogens) are optimal for identifying genetic resources for crop improvement.

The reactions of the 38 accessions to *Bgt* E09 are listed in Table 2. Remarkably, there were 18 AS genotypes susceptible to powdery mildew isolates of *Bgt* E09, while another 20 of ES genotypes were highly resistant at the seedling stage under the same controlled biological incubator conditions (Table 2). The same results were obtained under the controlled greenhouse conditions using a mixture of powdery mildew races at both seedling and adult stages. This is the first research on the phenotype and genotype adaptability of the resistance to powdery mildew of wild emmer between the abutting “African” slope and “European” slope in EC I. We have shown *inter*-slope adaptive and speciational patterns at ECII across phylogeny from bacteria, through soil fungi, plants and animals including mammals (Nevo list of "Evolution Canyon" publications at http://evolution.haifa.ac.il, including studies on wild emmer wheat and wild barley [1–17]). Our study on genetic diversity and the phenotype/genotype adaptability of the resistance to powdery mildew of wild emmer wheat locally at EC followed our regional analysis across Israel [44]. These regional and local studies are complementary. The uniqueness of the current study is its unfolding resistance locally across very close abutting slopes, separate, on average, by only 250 meters. Future studies could map the resistant genes, clone and transform them to cultivated crops thereby increasing world food production. We suggest that the EC microscale model offers an optimal opportunity to study the influence of microclimatic ecological divergence on the population genetics and genome evolution of natural populations. These studies could substantially support crop improvement and food production in a world whose population will reach 10 billions in 2050.

**Table 2:** The reactions of the 38 genotypes to *Bgt* E09, a prevailing powdery mildew pathotype in the Beijing area.

Accessions	Plant reaction^a^	Genotypes	Plant reaction^a^
**ES1**	R	**AS1**	S
**ES2**	R	**AS3**	S
**ES3**	R	**AS4**	S
**ES4**	R	**AS5**	S
**ES5**	R	**AS6**	S
**ES6**	R	**AS7**	S
**ES7**	R	**AS8**	S
**ES8**	R	**AS9**	S
**ES9**	R	**AS10**	S
**ES10**	R	**AS11**	S
**ES11**	R	**AS12**	S
**ES12**	R	**AS13**	S
**ES13**	R	**AS14**	S
**ES14**	R	**AS15**	S
**ES15**	R	**AS16**	S
**ES16**	R	**AS17**	S
**ES17**	R	**AS18**	S
**ES18**	R	**AS19**	S
**ES19**	R		
**ES20**	R		

^a^ Plant reaction: R resistant (IT = 0–2), S susceptible (IT = 3–4)
